# Supplementary material for: Mapping the Morbidity Risk Associated with Coal Mining in Queensland, Australia
Source: Int J Environ Res Public Health. 2022 Jan 21;19(3):1206. doi: 10.3390/ijerph19031206 (PMC8834562; doi:10.3390/ijerph19031206)
Supplement: Supplementary file 1 [file ijerph-19-01206-s001.zip › ijerph-1516230-supplementary.pdf]

Supplementary materials

Manuscript: Mapping the morbidity risk associated with coal mining in Queensland, Australia

Calculation of coal production per LGA

Spatial data of mining lease surface areas (i.e. shapefiles) containing coal mine name; mineral mined, mining lease tenure number and date of approval were obtained from the Queensland spatial catalogue [1]. Coal mining leases with date of approval until 2010 were used to produce a polygon shapefile of the coal mining areas for the study period. Additional data on coalmines provided by the Department of Natural Resources and Mines (DNRM), were incorporated to produce a single polygon shapefile of all coal mining areas. The data of coal production from open surface coal mines that included coalmine name and Gross Raw Output (GRO) (i.e. coal produced) per year were obtained from the DNRM [2]. To estimate the aggregated coal mining area-level production from 1996 to 2010, the coal mine names were matched with the coal mining areas, using the mining lease tenure number as well as the descriptors in the Queensland Coals reports prepared by the DNRM and the Bureau of Mining and Petroleum. The proportion of coal mining area in each LGA of the LGA-map was estimated using the intersection tool in ArcMAp to calculate aggregated coal production (in million tons) in each LGA.

Table S1. Summary statistics of the standardised (age-adjusted) rates of hospitalisation for each of the disease groups in females and males

	Disease group	ICD-10 code	Mean	SD	Min	Q1	Q3	Max
Females								
	Circulatory diseases	I00 - 199	32.20	10.40	7.10	25.40	36.70	79.80
	Respiratory diseases	J00 – J99	33.80	18.10	7.90	23.40	38.40	146.20
	Hypertensive diseases	I10 - I16	1.90	1.80	0.00	0.80	2.60	11.20
	Chronic respiratory diseases	J40 – J47	10.10	5.30	2.80	6.80	12.40	36.40
	Diabetes mellitus	E08 – E13	5.20	3.90	0.50	3.10	5.60	26.40
	Lung cancer	C34	0.90	0.70	0.00	0.70	1.10	5.70
Males								
	Circulatory diseases	I00 - 199	40.60	10.10	11.50	35.20	46.70	76.20
	Respiratory diseases	J00 – J99	36.70	17.60	8.40	27.00	44.20	143.20
	Hypertensive diseases	I10 - I16	1.10	1.00	0.00	0.50	1.30	5.50
	Chronic respiratory diseases	J40 – J47	11.50	5.90	2.30	7.60	14.10	34.90
	Diabetes mellitus	E08 – E13	5.30	3.00	0.50	3.70	5.90	18.50
	Lung cancer	C34	2.10	1.00	0.00	1.50	2.70	7.00

Notes. Rates expressed per 1000 habitants. Q1 and Q3: Percentiles 25 and 75, respectively. SD: Standard Deviation.

Sensitivity analysis

The BYM model incorporates priors on the log of the structured and unstructured effect precisions (s and u in the regression models). To assess the effect of the priors on the model estimates, the fit of the model was compared for the following priors:

$$Prior\ 1.\ \log \tau_v \sim \log Gamma\ (0.1, 0.01)\,, \log \tau_v \sim \log Gamma\ (0.1, 0.01)$$

$$Prior\ 2.\ \log \tau_v \sim \log Gamma\ (0.5, 0.001)\,, \log \tau_v \sim \log Gamma\ (0.5, 0.001)$$

$$Prior\ 3.\ \log \tau_v \sim \log Gamma\ (1, 0.005)\,, \log \tau_v \sim \log Gamma\ (1, 0.005)$$

The models were compared using the Deviance Information Criterion (DIC) that is based on the deviance statistic and the effective number of parameters of the model [3]. A model with a lower DIC value has a better fit. The models with each of the three priors found a positive association of coal production with the sHR for each of the disease groups after adjusting for sociodemographic and environmental factors. The model with prior 1 had the best fit for all disease groups except cancer of bronchus and lung, for which prior 3 produced the model with the best fit. The table below shows the comparison of the model fit using the 3 priors.

Table S2. Deviance Information Criterion of the regression models using each of the 3 priors, for each disease group

**Disease group**	**Prior 1**	**Prior 2**	**Prior 3**
Diseases circulatory system	**1443.022**	1451.961	1453.165
Diseases respiratory system	**1435.256**	1436.827	1437.559
Hypertensive diseases	**992.71**	993.217	993.6169
Chronic lower respiratory diseases	**1272.968**	1274.295	1274.477
Diabetes mellitus	**1161.664**	1162.722	1162.817
Cancer of bronchus and lung	938.5418	918.9173	**914.0862**

Table S3: LGA-specific relative risk (and 95% CI) of hospitalisation (compared to the whole of Queensland) (coal mining LGAs shaded in grey).

**LGA**	Circulatory diseases	Respiratory diseases	Cancer bronchus &lung	Diabetes mellitus	Chronic resp. diseases	Hypertensive diseases
Banana (S)	1.013 (0.972 - 1.055)	1.023 (0.953 - 1.097)	1.023 (0.944 - 1.109)	0.996 (0.907 - 1.092)	1.036 (0.958 - 1.117)	1.198 (1.002 - 1.42)
Bauhinia (S)	1.001 (0.939 - 1.065)	1.046 (0.96 - 1.137)	1.005 (0.918 - 1.098)	0.943 (0.806 - 1.089)	1.006 (0.89 - 1.13)	1.12 (0.839 - 1.45)
Belyando (S)	0.975 (0.89 - 1.066)	0.96 (0.79 - 1.155)	0.995 (0.906 - 1.091)	0.999 (0.832 - 1.189)	0.953 (0.8 - 1.127)	1.004 (0.684 - 1.425)
Bowen (S)	0.992 (0.946 - 1.04)	0.998 (0.908 - 1.094)	1.008 (0.924 - 1.097)	1.028 (0.924 - 1.139)	1.041 (0.948 - 1.139)	1.083 (0.854 - 1.35)
Broadsound (S)	0.989 (0.922 - 1.06)	1.134 (0.995 - 1.288)	0.978 (0.889 - 1.069)	0.92 (0.784 - 1.07)	1.063 (0.933 - 1.207)	1.161 (0.856 - 1.542)
Chinchilla (S)	1.024 (0.978 - 1.072)	0.962 (0.888 - 1.041)	0.999 (0.916 - 1.088)	1.013 (0.901 - 1.132)	0.966 (0.875 - 1.063)	1.136 (0.895 - 1.413)
Duaringa (S)	1.028 (0.97 - 1.089)	1.132 (1.023 - 1.25)	1.006 (0.921 - 1.097)	1.226 (1.088 - 1.38)	1.124 (1.01 - 1.248)	1.223 (0.952 - 1.548)

Emerald (S)	1.02 (0.979 - 1.063)	1.09 (1.023 - 1.159)	1.003 (0.921 - 1.091)	1.074 (0.975 - 1.179)	1.053 (0.976 - 1.135)	1.005 (0.81 - 1.226)
Ipswich (C)	1.005 (0.972 - 1.039)	1.021 (0.942 - 1.104)	1.017 (0.957 - 1.079)	1.003 (0.933 - 1.076)	1.034 (0.966 - 1.104)	0.815 (0.684 - 0.962)
Millmerran (S)	0.994 (0.936 - 1.053)	1.04 (0.935 - 1.152)	0.998 (0.908 - 1.095)	1.038 (0.904 - 1.185)	1.054 (0.945 - 1.171)	1.082 (0.797 - 1.428)
Murilla (S)	0.933 (0.88 - 0.987)	1.082 (0.981 - 1.189)	1.009 (0.918 - 1.107)	1.074 (0.933 - 1.229)	1.062 (0.955 - 1.175)	0.986 (0.767 - 1.242)
Nanango (S)	1.026 (0.98 - 1.073)	0.995 (0.914 - 1.08)	0.976 (0.897 - 1.057)	1.024 (0.921 - 1.135)	1.046 (0.958 - 1.138)	0.991 (0.777 - 1.24)
Nebo (S)	0.99 (0.916 - 1.069)	0.968 (0.848 - 1.1)	0.982 (0.887 - 1.08)	0.939 (0.787 - 1.106)	0.866 (0.737 - 1.005)	0.848 (0.573 - 1.188)
Peak Downs (S)	0.893 (0.821 - 0.967)	1.004 (0.868 - 1.155)	0.973 (0.877 - 1.071)	0.963 (0.807 - 1.138)	0.837 (0.712 - 0.974)	0.974 (0.671 - 1.36)
Rosalie (S)	0.986 (0.935 - 1.038)	0.975 (0.885 - 1.072)	0.981 (0.891 - 1.074)	0.942 (0.833 - 1.058)	1.01 (0.916 - 1.111)	0.751 (0.558 - 0.98)
Wambo (S)	0.983 (0.926 - 1.042)	0.928 (0.836 - 1.028)	1.007 (0.921 - 1.1)	0.944 (0.821 - 1.076)	0.968 (0.863 - 1.081)	0.822 (0.596 - 1.091)
Aramac (S)	0.919 (0.846 - 0.993)	0.984 (0.857 - 1.121)	0.995 (0.905 - 1.091)	0.99 (0.82 - 1.18)	0.933 (0.804 - 1.073)	0.947 (0.678 - 1.278)
Atherton (S)	1.02 (0.985 - 1.055)	1.033 (0.974 - 1.096)	1.012 (0.925 - 1.104)	1.057 (0.962 - 1.158)	1.056 (0.983 - 1.132)	1.031 (0.835 - 1.251)
Aurukun (S)	1.087 (0.963 - 1.225)	1.353 (1.026 - 1.746)	0.995 (0.855 - 1.15)	1.064 (0.823 - 1.335)	1.116 (0.868 - 1.41)	1.525 (0.814 - 2.597)
Balonne (S)	1.007 (0.959 - 1.056)	0.947 (0.857 - 1.04)	1.008 (0.922 - 1.1)	1.149 (1.029 - 1.278)	1.035 (0.947 - 1.127)	1.289 (1.024 - 1.596)
Barcaldine (S)	1.006 (0.939 - 1.075)	1.036 (0.929 - 1.152)	0.931 (0.837 - 1.024)	1.049 (0.895 - 1.218)	1.042 (0.92 - 1.174)	1.187 (0.851 - 1.596)
Barcoo (S)	0.978 (0.887 - 1.075)	1.04 (0.878 - 1.221)	0.999 (0.904 - 1.103)	1.014 (0.827 - 1.228)	1.036 (0.864 - 1.231)	1.05 (0.695 - 1.52)
Beaudesert (S)	1.009 (0.972 - 1.048)	0.966 (0.889 - 1.05)	1.015 (0.944 - 1.088)	0.949 (0.875 - 1.027)	0.925 (0.858 - 0.996)	0.903 (0.749 - 1.08)
Bendemere (S)	1.028 (0.949 - 1.113)	1.071 (0.955 - 1.197)	1.009 (0.912 - 1.114)	1.027 (0.852 - 1.224)	1.054 (0.906 - 1.216)	1.102 (0.735 - 1.572)
Biggenden (S)	1.011 (0.949 - 1.075)	1.035 (0.942 - 1.132)	0.996 (0.905 - 1.093)	1.031 (0.884 - 1.192)	1.055 (0.93 - 1.19)	1.121 (0.857 - 1.428)
Blackall (S)	0.919 (0.863 - 0.975)	0.954 (0.861 - 1.054)	0.992 (0.902 - 1.087)	1.014 (0.868 - 1.174)	0.882 (0.776 - 0.996)	0.957 (0.742 - 1.208)
Boonah (S)	1.008 (0.961 - 1.055)	0.989 (0.899 - 1.085)	1.006 (0.913 - 1.106)	0.975 (0.864 - 1.095)	1.033 (0.937 - 1.135)	0.91 (0.686 - 1.177)
Booringa (S)	1.009 (0.949 - 1.071)	1.068 (0.982 - 1.157)	1.006 (0.917 - 1.103)	1.072 (0.926 - 1.232)	1.033 (0.926 - 1.148)	1.184 (0.887 - 1.538)
Boulia (S)	1.028 (0.933 - 1.13)	1.159 (0.976 - 1.365)	1.011 (0.906 - 1.126)	1.137 (0.932 - 1.374)	1.103 (0.921 - 1.31)	1.075 (0.677 - 1.613)
Brisbane (C)	1.012 (0.96 - 1.065)	0.912 (0.804 - 1.03)	1.016 (0.952 - 1.082)	0.93 (0.836 - 1.03)	0.959 (0.865 - 1.06)	0.758 (0.595 - 0.95)
Bulloo (S)	0.965 (0.872 - 1.064)	1.03 (0.881 - 1.193)	0.998 (0.895 - 1.11)	0.946 (0.757 - 1.16)	0.997 (0.824 - 1.192)	0.943 (0.579 - 1.432)
Bundaberg (C)	0.895 (0.858 - 0.933)	1.022 (0.946 - 1.103)	0.989 (0.922 - 1.058)	1.018 (0.935 - 1.106)	1.031 (0.961 - 1.106)	1.186 (0.995 - 1.402)
Bungil (S)	0.993 (0.923 - 1.067)	0.998 (0.895 - 1.109)	0.993 (0.901 - 1.091)	0.961 (0.808 - 1.128)	1.027 (0.897 - 1.168)	1.133 (0.812 - 1.523)
Burdekin (S)	0.994 (0.957 - 1.032)	0.993 (0.92 - 1.072)	0.996 (0.912 - 1.084)	1.048 (0.96 - 1.141)	0.995 (0.92 - 1.075)	1.278 (1.062 - 1.524)
Burke (S)	1.03 (0.934 - 1.134)	0.841 (0.681 - 1.02)	1.007 (0.897 - 1.127)	0.957 (0.776 - 1.16)	1.07 (0.902 - 1.26)	0.609 (0.398 - 0.884)
Burnett (S)	0.938 (0.904 - 0.973)	0.675 (0.632 - 0.72)	0.988 (0.91 - 1.068)	0.851 (0.786 - 0.92)	0.766 (0.712 - 0.821)	0.666 (0.545 - 0.8)
Caboolture (S)	0.992 (0.962 - 1.022)	1.027 (0.966 - 1.091)	1.011 (0.955 - 1.069)	0.953 (0.897 - 1.012)	1.059 (1.004 - 1.116)	0.71 (0.61 - 0.82)
Cairns (C)	1.007 (0.974 - 1.041)	1.054 (0.978 - 1.135)	0.994 (0.933 - 1.057)	1.017 (0.945 - 1.092)	1.025 (0.959 - 1.094)	0.923 (0.783 - 1.081)
Calliope (S)	1.011 (0.976 - 1.047)	1.005 (0.949 - 1.063)	1.007 (0.929 - 1.09)	0.86 (0.779 - 0.945)	0.889 (0.823 - 0.959)	1.185 (0.998 - 1.393)
Caloundra (C)	1.005 (0.979 - 1.03)	0.925 (0.872 - 0.98)	1.021 (0.962 - 1.084)	0.945 (0.891 - 1.001)	0.957 (0.907 - 1.009)	0.813 (0.705 - 0.933)
Cambooya (S)	0.953 (0.893 - 1.014)	0.917 (0.821 - 1.022)	0.993 (0.899 - 1.092)	0.9 (0.776 - 1.035)	0.83 (0.725 - 0.943)	0.757 (0.531 - 1.034)
Cardwell (S)	1.02 (0.975 - 1.066)	1.047 (0.966 - 1.135)	1.001 (0.907 - 1.101)	1.074 (0.97 - 1.186)	1.079 (0.991 - 1.172)	0.806 (0.607 - 1.039)
Carpentaria (S)	1.065 (0.979 - 1.158)	1.219 (1.005 - 1.463)	1.013 (0.916 - 1.12)	1.397 (1.156 - 1.674)	1.123 (0.956 - 1.311)	1.587 (1.076 - 2.261)
Charters Towers (C)	1.02 (0.969 - 1.073)	1.126 (1.026 - 1.234)	1.013 (0.903 - 1.132)	1.153 (1.032 - 1.286)	1.13 (1.026 - 1.242)	1.417 (1.112 - 1.774)
Clifton (S)	1.023 (0.959 - 1.09)	1.071 (0.953 - 1.199)	0.992 (0.895 - 1.095)	1.01 (0.86 - 1.177)	1.02 (0.89 - 1.161)	1.05 (0.742 - 1.434)
Cloncurry (S)	1.07 (1.001 - 1.142)	1.073 (0.936 - 1.222)	1.024 (0.926 - 1.134)	1.203 (1.05 - 1.373)	0.975 (0.858 - 1.103)	0.981 (0.725 - 1.296)
Cook (S)	1.096 (1.028 - 1.169)	1.156 (1.005 - 1.322)	1.035 (0.932 - 1.15)	0.915 (0.794 - 1.048)	1.25 (1.108 - 1.41)	1.585 (1.178 - 2.09)
Cooloola (S)	1.016 (0.988 - 1.044)	0.985 (0.933 - 1.039)	1.019 (0.95 - 1.091)	1.034 (0.969 - 1.103)	0.984 (0.929 - 1.042)	0.899 (0.765 - 1.048)
Crow's Nest (S)	1.005 (0.956 - 1.056)	0.895 (0.81 - 0.986)	0.984 (0.895 - 1.075)	0.87 (0.768 - 0.98)	0.894 (0.805 - 0.99)	0.821 (0.621 - 1.057)
Croydon (S)	1.002 (0.902 - 1.108)	1.067 (0.87 - 1.292)	1.003 (0.899 - 1.115)	1.073 (0.864 - 1.315)	1.046 (0.857 - 1.262)	1.081 (0.651 - 1.678)
Dalby (T)	1.005 (0.957 - 1.055)	1.035 (0.927 - 1.15)	1.005 (0.897 - 1.12)	1.118 (0.999 - 1.247)	1.085 (0.985 - 1.192)	1.065 (0.806 - 1.375)
Dalrymple (S)	0.796 (0.727 - 0.864)	0.632 (0.544 - 0.726)	0.98 (0.892 - 1.072)	0.946 (0.806 - 1.097)	0.827 (0.713 - 0.948)	0.733 (0.499 - 1.006)
Diamantina (S)	0.968 (0.867 - 1.074)	1.064 (0.875 - 1.277)	0.999 (0.893 - 1.115)	1.065 (0.853 - 1.311)	1.047 (0.855 - 1.267)	1.059 (0.634 - 1.65)
Douglas (S)	1.015 (0.961 - 1.071)	1.083 (0.969 - 1.207)	1.007 (0.91 - 1.111)	1.128 (1.003 - 1.263)	1.107 (0.993 - 1.232)	1.434 (1.118 - 1.809)
Eacham (S)	1.028 (0.988 - 1.07)	1.035 (0.976 - 1.095)	1.013 (0.919 - 1.114)	1.024 (0.913 - 1.14)	1.049 (0.96 - 1.141)	1.019 (0.791 - 1.279)
Eidsvold (S)	1.037 (0.964 - 1.115)	1.218 (1.073 - 1.377)	1.004 (0.914 - 1.1)	1.106 (0.938 - 1.299)	1.093 (0.957 - 1.241)	1.13 (0.788 - 1.557)
Esk (S)	1.008 (0.97 - 1.047)	1.016 (0.942 - 1.095)	1.015 (0.937 - 1.097)	0.975 (0.886 - 1.069)	1.036 (0.96 - 1.116)	1.052 (0.856 - 1.277)
Etheridge (S)	1.005 (0.921 - 1.094)	0.926 (0.782 - 1.086)	0.999 (0.904 - 1.101)	0.993 (0.817 - 1.189)	0.941 (0.785 - 1.112)	1.028 (0.679 - 1.476)
Fitzroy (S)	1.008 (0.969 - 1.048)	1.048 (0.991 - 1.108)	1.001 (0.922 - 1.084)	0.984 (0.893 - 1.08)	0.937 (0.862 - 1.015)	0.929 (0.736 - 1.146)
Flinders (S)	0.972 (0.91 - 1.037)	1.043 (0.925 - 1.169)	1.001 (0.908 - 1.1)	1.044 (0.89 - 1.214)	1.061 (0.937 - 1.194)	1.241 (0.916 - 1.635)
Gatton (S)	1.017 (0.975 - 1.06)	0.966 (0.886 - 1.052)	1.005 (0.925 - 1.09)	0.924 (0.833 - 1.02)	0.955 (0.875 - 1.039)	0.899 (0.711 - 1.118)
Gayndah (S)	1.012 (0.958 - 1.068)	1.03 (0.955 - 1.108)	0.971 (0.886 - 1.056)	1.023 (0.902 - 1.154)	1.038 (0.933 - 1.15)	0.968 (0.769 - 1.196)
Gladstone (C)	1.013 (0.981 - 1.046)	1.068 (1.004 - 1.134)	1.016 (0.937 - 1.098)	0.96 (0.887 - 1.037)	1.009 (0.944 - 1.077)	1.195 (1.015 - 1.394)
Gold Coast (C)	1.018 (0.983 - 1.053)	0.904 (0.83 - 0.984)	1.014 (0.963 - 1.067)	0.938 (0.874 - 1.004)	0.9 (0.839 - 0.965)	0.762 (0.645 - 0.893)
Goondiwindi (T)	0.981 (0.929 - 1.034)	0.941 (0.838 - 1.05)	0.993 (0.881 - 1.112)	1.023 (0.897 - 1.159)	0.927 (0.832 - 1.027)	1.313 (1.009 - 1.67)
Herberton (S)	0.999 (0.954 - 1.045)	1.09 (1.007 - 1.177)	1.015 (0.927 - 1.111)	1.104 (0.988 - 1.229)	1.096 (0.999 - 1.199)	1.165 (0.903 - 1.472)
Hervey Bay (C)	1.018 (0.994 - 1.043)	0.914 (0.871 - 0.958)	1.019 (0.957 - 1.083)	0.98 (0.924 - 1.039)	0.956 (0.908 - 1.007)	0.751 (0.643 - 0.868)
Hinchinbrook (S)	0.984 (0.943 - 1.025)	1.163 (1.073 - 1.257)	1.019 (0.934 - 1.11)	1.151 (1.053 - 1.253)	1.163 (1.078 - 1.253)	1.284 (1.033 - 1.573)
Ilfracombe (S)	0.987 (0.895 - 1.085)	1.047 (0.878 - 1.237)	0.991 (0.894 - 1.093)	0.967 (0.783 - 1.175)	0.969 (0.799 - 1.161)	0.969 (0.615 - 1.441)
Inglewood (S)	1.019 (0.95 - 1.091)	1.057 (0.919 - 1.208)	1.004 (0.904 - 1.112)	1.098 (0.932 - 1.284)	0.936 (0.82 - 1.061)	1.103 (0.795 - 1.488)
Isis (S)	1.03 (0.982 - 1.079)	0.923 (0.859 - 0.991)	1.013 (0.925 - 1.108)	0.923 (0.816 - 1.037)	0.99 (0.9 - 1.084)	1.141 (0.908 - 1.408)
Isisford (S)	0.973 (0.878 - 1.073)	0.953 (0.78 - 1.15)	0.991 (0.894 - 1.095)	1.014 (0.823 - 1.235)	0.971 (0.801 - 1.164)	0.982 (0.634 - 1.447)
Jericho (S)	0.962 (0.892 - 1.034)	0.966 (0.858 - 1.083)	0.988 (0.898 - 1.083)	0.997 (0.838 - 1.173)	0.966 (0.837 - 1.107)	1.058 (0.785 - 1.386)
Johnstone (S)	1.016 (0.978 - 1.055)	1.056 (0.977 - 1.139)	1.017 (0.936 - 1.103)	1.099 (1.009 - 1.193)	1.083 (1.006 - 1.165)	1.241 (1.024 - 1.488)
Jondaryan (S)	1.013 (0.966 - 1.062)	1.03 (0.936 - 1.131)	0.976 (0.889 - 1.065)	0.983 (0.88 - 1.093)	1.009 (0.918 - 1.107)	0.833 (0.639 - 1.063)
Kilcoy (S)	1.019 (0.965 - 1.075)	0.986 (0.903 - 1.074)	1.011 (0.919 - 1.109)	0.931 (0.805 - 1.067)	1.022 (0.917 - 1.134)	0.965 (0.712 - 1.27)
Kilkivan (S)	1.035 (0.978 - 1.095)	0.975 (0.896 - 1.059)	1.008 (0.922 - 1.101)	0.981 (0.857 - 1.116)	1.002 (0.895 - 1.116)	0.97 (0.724 - 1.265)
Kingaroy (S)	1.022 (0.984 - 1.062)	1.031 (0.961 - 1.105)	0.999 (0.915 - 1.089)	1.079 (0.983 - 1.182)	1.061 (0.984 - 1.142)	1.139 (0.928 - 1.378)
Kolan (S)	1.02 (0.969 - 1.073)	0.991 (0.915 - 1.072)	1.006 (0.918 - 1.1)	0.969 (0.852 - 1.096)	1.032 (0.934 - 1.138)	1.033 (0.787 - 1.323)

Laidley (S)	1.012 (0.968 - 1.057)	1.067 (0.974 - 1.166)	1.013 (0.929 - 1.101)	1.105 (0.997 - 1.223)	1.099 (1.005 - 1.199)	1.018 (0.804 - 1.267)
Livingstone (S)	1.032 (1.003 - 1.061)	1.024 (0.974 - 1.077)	1.016 (0.948 - 1.088)	1.015 (0.948 - 1.087)	0.989 (0.932 - 1.049)	0.935 (0.792 - 1.092)
Logan (C)	1.004 (0.95 - 1.061)	0.957 (0.845 - 1.079)	1.012 (0.944 - 1.084)	0.933 (0.838 - 1.035)	0.959 (0.864 - 1.06)	0.703 (0.548 - 0.886)
Longreach (S)	1.002 (0.946 - 1.059)	1.07 (0.975 - 1.17)	1.002 (0.91 - 1.099)	1.063 (0.927 - 1.213)	1.088 (0.977 - 1.207)	1.094 (0.81 - 1.434)
Mackay (C)	1.012 (0.986 - 1.039)	1.043 (0.99 - 1.098)	1.009 (0.951 - 1.069)	1.052 (0.994 - 1.112)	1.01 (0.96 - 1.063)	1.082 (0.952 - 1.225)
Mareeba (S)	1.02 (0.976 - 1.065)	1.129 (1.023 - 1.24)	1.029 (0.947 - 1.118)	1.12 (1.018 - 1.227)	1.141 (1.049 - 1.239)	1.374 (1.107 - 1.681)
Maroochy (S)	1.014 (0.99 - 1.039)	0.882 (0.832 - 0.934)	1.015 (0.962 - 1.07)	0.855 (0.808 - 0.904)	0.907 (0.862 - 0.954)	0.696 (0.61 - 0.791)
Maryborough (C)	0.968 (0.94 - 0.995)	1.009 (0.963 - 1.056)	1.007 (0.934 - 1.082)	1.011 (0.944 - 1.081)	1.042 (0.988 - 1.098)	1.106 (0.955 - 1.271)
McKinlay (S)	1.045 (0.96 - 1.136)	1.149 (0.993 - 1.322)	1.013 (0.914 - 1.122)	0.988 (0.802 - 1.197)	1.125 (0.96 - 1.309)	1.209 (0.839 - 1.679)
Mirani (S)	1.03 (0.981 - 1.081)	1.004 (0.927 - 1.085)	1.007 (0.91 - 1.11)	1.009 (0.888 - 1.14)	0.992 (0.891 - 1.099)	0.984 (0.727 - 1.288)
Miriam Vale (S)	1.016 (0.959 - 1.075)	0.842 (0.764 - 0.925)	1.006 (0.91 - 1.11)	0.903 (0.784 - 1.032)	0.859 (0.759 - 0.967)	1.034 (0.767 - 1.35)
Monto (S)	1.022 (0.965 - 1.081)	1.056 (0.978 - 1.137)	1.005 (0.914 - 1.103)	0.971 (0.839 - 1.115)	1.037 (0.923 - 1.159)	1.101 (0.812 - 1.441)
Mornington (S)	0.909 (0.809 - 1.015)	0.393 (0.296 - 0.506)	1.007 (0.864 - 1.167)	0.757 (0.594 - 0.928)	0.623 (0.501 - 0.755)	0.386 (0.224 - 0.607)
Mount Isa (C)	1.134 (1.058 - 1.214)	1.26 (1.08 - 1.463)	1.044 (0.942 - 1.159)	1.355 (1.176 - 1.561)	1.265 (1.109 - 1.444)	1.344 (0.966 - 1.828)
Mount Morgan (S)	0.99 (0.935 - 1.048)	1.078 (0.975 - 1.188)	0.967 (0.871 - 1.067)	1.012 (0.887 - 1.147)	0.988 (0.886 - 1.097)	1.322 (1.006 - 1.695)
Mundubbera (S)	1.017 (0.959 - 1.077)	1.065 (0.985 - 1.149)	0.998 (0.904 - 1.099)	1.019 (0.877 - 1.175)	1.059 (0.948 - 1.176)	1.176 (0.886 - 1.522)
Murgon (S)	0.956 (0.907 - 1.005)	0.771 (0.678 - 0.87)	1.013 (0.913 - 1.122)	0.786 (0.691 - 0.885)	0.93 (0.841 - 1.022)	1.008 (0.806 - 1.241)
Murweh (S)	1.001 (0.957 - 1.046)	1.054 (0.989 - 1.121)	1.006 (0.92 - 1.098)	1.08 (0.959 - 1.209)	1.024 (0.942 - 1.109)	1.265 (1.042 - 1.513)
Noosa (S)	1.016 (0.99 - 1.042)	0.849 (0.802 - 0.899)	1.019 (0.95 - 1.091)	0.845 (0.788 - 0.905)	0.867 (0.817 - 0.92)	0.812 (0.697 - 0.94)
Paroo (S)	0.838 (0.783 - 0.893)	0.879 (0.766 - 1.001)	1.007 (0.915 - 1.105)	0.837 (0.723 - 0.959)	0.76 (0.657 - 0.87)	0.714 (0.517 - 0.951)
Perry (S)	1.044 (0.953 - 1.142)	1.078 (0.92 - 1.254)	1.002 (0.907 - 1.105)	0.996 (0.817 - 1.203)	1.016 (0.863 - 1.187)	1.111 (0.751 - 1.577)
Pine Rivers (S)	0.972 (0.937 - 1.008)	0.928 (0.856 - 1.004)	0.981 (0.919 - 1.045)	0.898 (0.834 - 0.966)	0.94 (0.877 - 1.007)	0.552 (0.46 - 0.657)
Pittsworth (S)	1.015 (0.96 - 1.071)	1.043 (0.941 - 1.153)	0.985 (0.887 - 1.087)	1.01 (0.883 - 1.149)	1.031 (0.922 - 1.148)	1.218 (0.918 - 1.583)
Quilpie (S)	1.004 (0.933 - 1.08)	0.966 (0.868 - 1.073)	0.909 (0.908 - 1.099)	0.977 (0.818 - 1.155)	0.926 (0.819 - 1.041)	1.124 (0.831 - 1.478)
Redcliffe (C)	1.008 (0.921 - 1.1)	0.958 (0.773 - 1.172)	1.009 (0.915 - 1.109)	0.896 (0.745 - 1.065)	0.992 (0.83 - 1.174)	0.785 (0.516 - 1.141)
Redland (S)	1.021 (0.99 - 1.053)	0.963 (0.895 - 1.037)	1.029 (0.967 - 1.092)	0.969 (0.906 - 1.035)	1.01 (0.947 - 1.076)	0.904 (0.773 - 1.051)
Richmond (S)	0.982 (0.907 - 1.061)	1.084 (0.94 - 1.242)	0.995 (0.898 - 1.099)	1.013 (0.838 - 1.209)	1.043 (0.901 - 1.2)	1.223 (0.845 - 1.707)
Rockhampton (C)	1.015 (0.986 - 1.044)	1.046 (0.987 - 1.107)	0.977 (0.919 - 1.037)	1.07 (1.006 - 1.137)	1.021 (0.966 - 1.079)	1.129 (0.981 - 1.293)
Roma (T)	1.031 (0.985 - 1.079)	1.064 (0.978 - 1.155)	1.008 (0.902 - 1.121)	1.178 (1.056 - 1.309)	1.082 (0.987 - 1.182)	1.257 (0.977 - 1.582)
Sarina (S)	0.996 (0.956 - 1.036)	1.103 (1.035 - 1.175)	1.009 (0.92 - 1.104)	1.074 (0.977 - 1.177)	1.113 (1.032 - 1.199)	1.113 (0.89 - 1.366)
Stanthorpe (S)	1.039 (0.958 - 1.125)	0.96 (0.782 - 1.166)	0.995 (0.889 - 1.109)	1.045 (0.878 - 1.235)	0.981 (0.83 - 1.151)	1.249 (0.832 - 1.812)
Tambo (S)	0.994 (0.914 - 1.079)	1.013 (0.899 - 1.134)	0.998 (0.904 - 1.099)	1.011 (0.84 - 1.203)	0.993 (0.847 - 1.155)	1.027 (0.681 - 1.472)
Tara (S)	0.997 (0.941 - 1.056)	1.051 (0.95 - 1.159)	1.011 (0.924 - 1.105)	1.034 (0.906 - 1.173)	1.043 (0.937 - 1.157)	1.142 (0.887 - 1.444)
Taroom (S)	1.03 (0.97 - 1.093)	1.053 (0.969 - 1.14)	1.007 (0.922 - 1.098)	0.994 (0.858 - 1.14)	1.005 (0.894 - 1.124)	1.197 (0.936 - 1.499)
Thuringowa (C)	0.975 (0.94 - 1.011)	0.977 (0.906 - 1.054)	0.965 (0.894 - 1.039)	0.993 (0.92 - 1.071)	0.899 (0.836 - 0.966)	0.565 (0.458 - 0.685)
Tiaro (S)	1.012 (0.953 - 1.073)	0.911 (0.824 - 1.004)	1.013 (0.92 - 1.112)	0.917 (0.798 - 1.047)	0.986 (0.879 - 1.101)	0.847 (0.613 - 1.128)
Toowoomba (C)	1.01 (0.952 - 1.07)	0.981 (0.852 - 1.123)	0.986 (0.915 - 1.06)	1 (0.886 - 1.122)	0.97 (0.863 - 1.085)	0.949 (0.719 - 1.226)
Torres (S)	1.084 (0.994 - 1.182)	1.413 (1.147 - 1.721)	0.976 (0.847 - 1.117)	0.816 (0.675 - 0.964)	1.305 (1.102 - 1.535)	1.807 (1.184 - 2.634)
Townsville (C)	1.013 (0.981 - 1.046)	0.995 (0.924 - 1.07)	0.989 (0.926 - 1.054)	1.093 (1.02 - 1.171)	0.995 (0.933 - 1.062)	0.679 (0.569 - 0.803)
Waggamba (S)	0.98 (0.914 - 1.048)	1.014 (0.913 - 1.122)	0.987 (0.891 - 1.087)	0.903 (0.762 - 1.057)	1.056 (0.936 - 1.187)	1.066 (0.763 - 1.435)
Warroo (S)	1.014 (0.939 - 1.093)	1.021 (0.906 - 1.144)	1.003 (0.91 - 1.103)	1.027 (0.858 - 1.215)	0.897 (0.78 - 1.024)	1.14 (0.831 - 1.514)
Warwick (S)	1.023 (0.974 - 1.075)	1.005 (0.893 - 1.128)	0.986 (0.902 - 1.073)	1.029 (0.921 - 1.146)	1.045 (0.945 - 1.152)	1.138 (0.882 - 1.446)
Whitsunday (S)	1.005 (0.963 - 1.048)	0.99 (0.912 - 1.073)	0.976 (0.883 - 1.072)	0.871 (0.781 - 0.966)	0.974 (0.891 - 1.061)	1.062 (0.852 - 1.304)
Winton (S)	0.967 (0.902 - 1.034)	1.101 (0.97 - 1.243)	1.008 (0.916 - 1.108)	1.04 (0.88 - 1.216)	1.023 (0.899 - 1.158)	1.036 (0.774 - 1.351)
Wondai (S)	1.034 (0.983 - 1.088)	1.064 (0.991 - 1.141)	0.987 (0.904 - 1.072)	1.057 (0.939 - 1.184)	1.095 (0.995 - 1.203)	1.063 (0.813 - 1.355)
Woocoo (S)	0.922 (0.86 - 0.985)	0.694 (0.62 - 0.772)	0.988 (0.897 - 1.082)	0.988 (0.861 - 1.127)	0.843 (0.733 - 0.959)	0.781 (0.551 - 1.052)

References

1. Queensland Government. (2019, March 22). Queensland spatial cataolgue - QSpatial. Retrieve from http://qldspatialinformationqldgovau/catalogue/custom/indexpage. 2019.
2. DNRM. (2015, Feburary 1). Practice Direction: Submission of statistical data relating to coal production and sales. Retrieved from https://wwwdnrmqldgovau/__data/assets/pdf_file/0009/238743/guide-completing-statistical-forms-coal-productionpdf. 2015.
3. Zhu L, Carlin BP. Comparing hierarchical models for spatio-temporally misaligned data using the deviance information criterion. Statistics in Medicine. 2000;19(17-18):2265-78. https//doi.org/https://doi.org/10.1002/1097-0258(20000915/30)19:17/18<2265::AID-SIM568>3.0.CO;2-6
